# Gene Expression Differences between Enriched Normal and Chronic Myelogenous Leukemia Quiescent Stem/Progenitor Cells and Correlations with Biological Abnormalities

**DOI:** 10.1155/2011/798592

**Published:** 2011-02-23

**Authors:** M. Affer, S. Dao, C. Liu, A. B. Olshen, Q. Mo, A. Viale, C. L. Lambek, T. G. Marr, B. D. Clarkson

**Affiliations:** ^1^Mayo Clinic, Scottsdale, AZ 85259, USA; ^2^Molecular Pharmacology & Chemistry Program, Sloan-Kettering Institute, Memorial Sloan-Kettering Cancer Center, New York, NY 10065, USA; ^3^Department of Epidemiology-Biostatistics UCSF, San Francisco, CA 94107, USA; ^4^Department of Epidemiology-Biostatistics, Sloan-Kettering Institute, Memorial Sloan-Kettering Cancer Center, New York, NY 10065, USA; ^5^Genomics Core Laboratory, Sloan-Kettering Institute, Memorial Sloan-Kettering Cancer Center, New York, NY 10065, USA; ^6^Hiberna Corporation, 1941 Pearl Street, Suite 200, Boulder, CO 80302, USA

## Abstract

In comparing gene expression of normal and CML CD34+ quiescent (G0) cell, 292 genes were downregulated and 192 genes upregulated in the CML/G0 Cells. The differentially expressed genes were grouped according to their reported functions, and correlations were sought with biological differences previously observed between the same groups. The most relevant findings include the following. (i) CML G0 cells are in a more advanced stage of development and more poised to proliferate than normal G0 cells. (ii) When CML G0 cells are stimulated to proliferate, they differentiate and mature more rapidly than normal counterpart. (iii) Whereas normal G0 cells form only granulocyte/monocyte colonies when stimulated by cytokines, CML G0 cells form a combination of the above and erythroid clusters and colonies. (iv) Prominin-1 is the gene most downregulated in CML G0 cells, and this appears to be associated with the spontaneous formation of erythroid colonies by CML progenitors without EPO.

## 1. Introduction

Chronic Myelogenous Leukemia (CML) represents about 15–20% of all cases of adult leukemia in western populations [1]. CML is a clonal myeloproliferative disease characterized by the presence in >95% of patients of the t(9;22)(q34;11) translocation known as the Philadelphia Chromosome [2]. This translocation causes expression of the BCR-ABL fusion protein, a tyrosine kinase with constitutively increased kinase activity [3] which is thought to be necessary and sufficient for the initiation of CML [4].

The development of small molecule tyrosine kinase inhibitors (TKIs), such as Imatinib Mesylate, which inhibits the increased BCR-ABL tyrosine kinase activity, have dramatically improved the prognosis of patients with CML [5, 6]. Imatinib Mesylate induces complete hematologic remissions and cytogenetic responses in the majority of patients in chronic phase CML, but the response in more advanced stages is usually only partial and less durable. About 4-5% of patients per year develop resistance to Imatinib either because of Bcr-Abl gene amplification or more commonly point mutations in Bcr-Abl [7, 8]. If Imatinib is discontinued because of toxicity or other reasons, the majority of patients relapse fairly promptly [9]. One hypothesis that could explain the customary relapses after stopping therapy is that TKIs and conventional cytotoxic drugs therapies are unable to eliminate all BCR-ABL positive cells, presumably sparing a relatively small number of leukemic early progenitors and stem cells that are quiescent and are not killed by the inhibitors or other drugs at clinically tolerable concentrations [10]. These cells constitute a reservoir of BCR/ABL positive cells capable of functioning as leukemic stem cells or “limited stem cells” [11]. If for any reason a patient has to stop therapy, these cells have sufficient self-renewal ability to recreate the disease by reconstituting the stem cell population and also enhancing the probability that the leukemic cells will develop resistance to the drugs, leaving bone marrow transplantation as the only option for survival.

In order to search for critical differences in biochemical pathways and regulatory networks between CML and normal quiescent progenitors and stem cells that might ultimately serve as selective targets, we decided to compare the gene expression profiles of the normal and CML quiescent cell fractions. As a strategy to enrich for these quiescent cells, we isolated CD34+ cells by positive selection from the mononuclear cells of normal bone marrow and CML blood samples and then separated the CD34+ cells into G0 and G_1_/S/G_2_/M fractions by flow cytometry. We found 1, 204 genes significantly upregulated and 1, 133 downregulated in CML-G0 cells compared to normal G0 cells (resp., 3.1% and 2.9% of the genes represented on the Affymetrix chip), thereby permitting us to compare gene expression profiles in highly enriched normal and leukemic quiescent progenitors and stem cells. 

## 2. Material and Methods

### 2.1. Patients and Bone Marrow Controls

A total number of eight patients diagnosed with Ph+ CML (three in accelerated phase and five in chronic phase) were used in this study. The CML samples were all obtained from patients hospitalized at the Memorial Hospital during the period 1990–2006.

The bone marrow samples were purchased from Cambrex (Cambrex Bio Science Rockland, Inc., Rockland, Maine 04841, USA).

### 2.2. CD34+ Cells Enrichment

Mononuclear cells were isolated on a Ficoll gradient (Ficoll-Paque PLUS, GE Healthcare, Cat# 17-1440-03) from total nucleated cells of bone marrows of four healthy donors and peripheral blood of eight patients with untreated Ph+ CML (five in chronic and three in early accelerated phase with 8.5–18% blasts in the peripheral blood). In the case of the patient samples, total cells after Ficoll were frozen in liquid nitrogen in RPMI plus 10% dimethyl sulfoxide and 10% fetal calf serum and stored until selection. The normal bone marrows were instead processed the same day of their arrival.

CD34+ cells were positively selected using a midiMACS immunomagnetic separation Kit (Miltenyi Biotec, Bergisch Gladbach, Germany, CD34 progenitor Cell Isolation Kit, Cat# 130-0460701) after one round of purification, the recovered cells were passed through another round of purification using a second column. This way the purity of the CD34+ recovered cells was 96–100% as assessed by flow cytometry. For the statistics about the starting number of cells from each sample and the CD34 recovered fraction, see Table 1. 

### 2.3. Hoechst 33342 and Pyronin Y Staining for G0 Cells Enrichment

CD34+ cells were resuspended at a concentration of two million per 0.5 ml of staining buffer (SB: HBSS without NaHCo_3_, 10 mM Hepes, 1% BSA, 2% Fetal Calf Serum) plus Hoechst 33342 (bisBenzimide H 33342, Cat# B2261, Sigma, St. Louis, Missouri, USA) at a final concentration of 20 nM/ml and incubated for 45′ in a water bath at 37^0^C. After washing once with SB plus 10% Hoechst, the cells were resuspended in 0.5 ml of SB plus Pyronin Y (Cat# P9172, Sigma) at a final concentration of 1 *μ*g/ml and kept in a water bath at 37°C for 20′, washed once and resuspended in 1 ml of SB.

**Figure 1:**
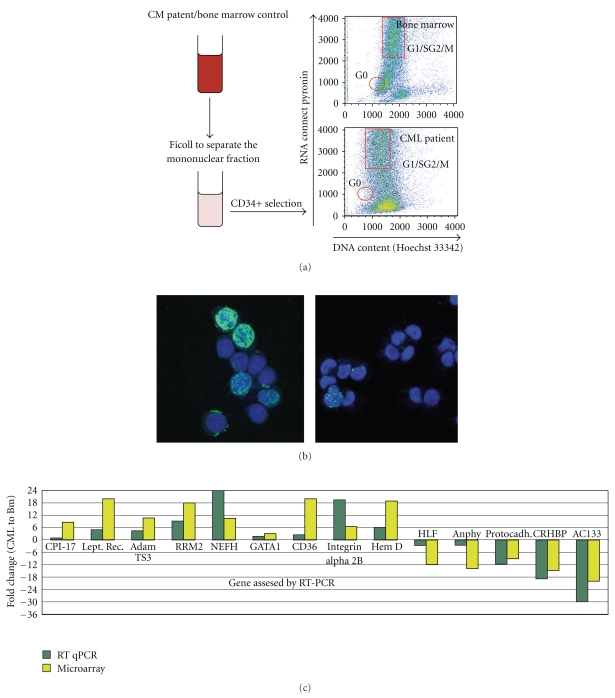
(a) Flow chart of the separation and sorting of the cells used for generating the microarray data. Cells from healthy bone marrows and CML patients went through a round of Percoll (to separate the mononuclear cells) and Ficoll (to eliminate dead cells). Highly enriched CD34+ cells were isolated from the mononuclear cells by two rounds of immunomagnetic separation using a Myltenyi kit, and the CD34+ cells were then stained it with Hoechst and Pyronin and sorted by FACS. The G0 cells are in the region of low fluorescence intensity for both dyes, and the G1/S/G2/M cells have a high level of Hoechst and PyroninY. (b) BrdU staining of a CML CD34+/G1/S/G2/M and CD34+/G0 quiescent fraction. To further validate our separation procedure, we pulsed labeled CML CD34+/G0 and G_1_/S/G_2_/M cells with BrdU. After 1 hour's exposure, 22% of the cells from the CML cycling fraction were labeled with BrdU compared to 0.68% of the quiescent cells. The BrdU positive cells are fluorescent (green) while the blue are Dapi stained nuclei without BrdU incorporation. Left panel: CML G_1_/S/G_2_/M cells. Right panel: CML G0 cells. (c) Gene expression array data validation by qPCR. To confirm the differences in gene expression found by our microarray data analysis between the CML/G0 and BM/G0, we performed an RT-qPCR on nine up- and five downregulated genes. Thirteen out of fourteen genes (the exception being CPI-17) have been confirmed to be up-, or downregulated by this second technique. For each sample, we ran four reactions for each gene and the average value has been used for the calculation of relative expression using the ΔΔct method and GAPDH as a calibrator. *y*-axis: gene expression fold change: CML relative to bone marrow. *x*-axis: gene tested by RT-qPCR.

After being stained, the cells were sorted using a MoFlo flow cytometer (DakoCytomation, Dako Colorado, Inc. Fort Collins, Colorado, USA) applying a gate in the region of logarithmic fluorescence intensity of 1000 + /−100 for both Pyronin Y (FL3) and Hoechst (FL7). An example of the gating strategies is shown in Figure 1(a).

### 2.4. BrdU Staining

Approximately 50,000 cells from the CML/G0 and CD34+/G1/S/G2/M fractions were analyzed for BrdU incorporation using a Roche kit (Cat# 11 296 736 001). Cells were pulsed with BrdU for an hour in complete growth medium, fixed with Ethanol, stained with a mouse anti-BrdU monoclonal antibody, and then incubated with an anti-mouse-Ig fluorescein conjugated antibody. Cells that incorporated BrdU were then counted using an immunofluorescence microscope Figure 1(b).

### 2.5. RNA Isolation, Labeling, Hybridization, and Microarray

Total RNA was isolated from each group of cells sorted from the G0 control fraction (BM) or CML sample using TRIzol reagent (Cat# 15596-026, Invitrogen Corp., Carlsbad, CA, USA). Quality of RNA was ensured before labeling by analyzing 5 pg of each individual sample using the RNA 6000 picoAssay and a Bioanalyzer 2100 (Agilent Technologies, Inc., Santa Clara, CA, USA). Samples with a RNA Integrity Number (RIN) greater than 7.0 were considered suitable for labeling. For each sample meeting this standard, 20 ng of total its RNA were labeled using the GeneChip two-cycle target labeling kit (Affymetrix, Inc., Santa Clara, CA, USA). Ten micrograms of labeled and fragmented cRNA were then individually hybridized to the Human Genome U133 plus 2.0 array (Affymetrix) at 45°C for 16 h. Automated washing and staining were performed using the Affymetrix Fluidics Station 400 according to the manufacturer's protocols. Finally, chips were scanned with a high-numerical Aperture and flying objective (FOL) lens in the GS3000 scanner (Affymetrix). In summary, from each sample (CML or Bone Marrow), we extracted RNA and performed a separate gene expression profile and the changes in gene expression were derived from the statistical analysis in which we compared CML samples (eight independent gene expression data) Versus Bone marrow (five independent gene expression data).

### 2.6. Statistical Analysis

The microarray data were quantile-normalized, and the gene expression values were estimated using the RMA method [12]. A linear regression model was used to model the gene expression values, in which a batch factor was added to the model to account for potential batch effect since arrays were run in two distinct batches two years apart. Differences between the G0 gene expression values of CML and normal samples were tested using the moderated *t*-statistics. Storey's *q*-value that control false discovery rate was used to correct for multiple hypothesis testing [13]. A *q*-value less than 0.1 was considered statistically significant. 

The microarray data have been deposited on the GEO public repository (http://www.ncbi.nlm.nih.gov/geo/) under accession no. 15881331.

### 2.7. RT-qPCR

RT-qPCR assay was used to determine the level of expression of the genes we found up and downregulated on the microarray.

We used the IScript One-Step RT-PCR Kit with SYBR Green (Cat# 170-8892, Biorad, Hercules, CA, USA) with the following conditions: 10^1^ @ 50°C, 5^1^ @ 95°C, 10^1^ @ 95°C, and 30^1^ @ 60°C using 1 ng of RNA for each reaction as a template. All samples were run in quadruplicate on an ABI 9700 platform (Perkin Elmer/Applied Biosystems, Foster City, CA, USA).

The relative expression of each gene was calculated using the ΔΔct method using GAPDH as a reference gene. Forward and reverse primers were designed in different exons, in order to avoid DNA contribution to our final PCR product, using Primer 3 software (available on line at: http://frodo.wi.mit.edu/).

For the sequence of the primers used, see Table 1 in Supplementary Material available online at doi:10.1155/2011/798592.

### 2.8. Colony Assays and Cytokines

Three or four hundred total CD34+ cells or CD34+ G0 and G_1_/S/G_2_/M enriched cells per plate were assayed for colony growth in 1.3% methylcellulose as described in detail previously [14].The cloning efficiency (C.E.) values are the average of 4 plates counted for each bar shown. Unless otherwise stated, either single (100 ng/ml) or three early acting cytokines: KL, FL, TPO (50 ng/ml each) with or without additional cytokines as noted: G-CSF, GM-CSF (10 ng/ml each), IL-3, IL-6 (20 ng/ml each), and EPO 1 IU/ml were added ± the drugs shown to the cultures to stimulate or inhibit colony growth. Unless otherwise noted, 3 cytokines refer to KL+FL+TPO and 5, 6, 7, or 8 cytokines refer to KL + FL + TPO + G-CSF + GM-CSF ± IL-3 ± IL-6 ± EPO in that order at the concentrations indicated above. Quadruplicate plates of each sample were counted at days 14 or 15 using an inverted microscope including estimates of colony lineage and size. A standardized scale and colorcode for estimating colony size has long been used in our laboratory which has been verified by plucking single large and multiple smaller (pooled) colonies and hemocytometer counts of the numbers of cells contained in the different sized colonies. GFU-GM: tiny <40 cells, small 40–100, medium 1000–10,000, large 10,000–40,000, X-large 40,000–100,000, XX-large > 100,000 cells; CFU-E and BFU-E and mixed: tiny < 50–5000, medium 5000–50,000, large 50,000–10^5^, X-large 10^5^–5 × 10^5^, XX-large > 5 × 10^5^ cells. G-CSF, GM-CSF, and IL-3 were obtained as gifts from Kirin Brewery Co., Gunma, Japan and KL (Kit ligand or SCF, Stem Cell Factor), FL (Flt3 ligand), TPO (Thrombopoietin), IL-6, and EPO (Erythropoietin) were purchased from R & D systems, Inc. CD 133/2 APC (293 C3) was purchased from Miltenyi Biotech, Gladback, Germany. Cord Blood samples were obtained from the New York Blood Center as samples judged too small for clinical use; Three or 4 samples were pooled for our studies.

## 3. Results

### 3.1. Custom

Defrosted mononuclear peripheral blood cells from eight CML patient samples or five fresh normal bone marrows were individually used to isolate the CD34+ fraction using the midiMACS immunomagnetic separation Kit from Miltenyi (see Material and Methods) with a percentage of purity of recovered cells varying between 95–100%.

CD34+ cells from each samples were subsequently stained with Hoechst 33342 and Pyronin Y and sorted individually according to their dye content (Figure 1(a)) [15]. The region chosen for sorting the quiescent fraction (G0) allowed us to avoid collecting dead cells and cross-contamination with cycling cells.

We also sorted the proliferating fraction (G_1_/S/G_2_/M), but due to its high heterogeneity, we did not conduct a detailed analysis on this fraction for comparison with the CD34+/G0 cells.

### 3.2. Statistics of Recovered Cells

Table 1 summarizes the numbers and percentage of CD34+/G0 cells and CD34+/G1/S/G2/M recovered from each patient and normal bone marrow.

After Percoll and Ficol separation of total blood cells from normal bone marrow or CML blood on average 22% of normal and 11% of CML mononuclear cells were recovered. After passing the MNCs twice on Miltenyi columns for positive selection of CD34+ cells, 3.06% of normal and 3.15% of CML highly enriched CD34+ cells were obtained from the MNC fractions. Due to the low quality of RNA recovered, one BM sample (#2) was not used to generate microarray data.

The CD34+ cells were further separated into proliferating (G1/S/G2/M) and quiescent (G0) fractions by flow cytometer using Hoechst and Pyronin y staining.

After sorting, the mean and range of recoveries of normal and CML CD34+/G1/S/G2/M were 18% and 14.7%, respectively, while the recoveries of normal and CML CD34+/G0 cells were 4.3% and 3.05%, respectively, or an average of 0.016 and 0.013%, respectively, of the total normal and CML starting cell populations.

### 3.3. Quiescent or Proliferative Status of G0 and G1/S/G2/M Cells

CD34+/G0 and G1/S/G2/M cells were pulse labeled with BrdU. An average of 27% (22–32%) of unstimulated CML CD34+ G1/S/G2/M cells incorporated BrdU after an incubation period of one hour while less than 1% of CML or normal CD34+ G0 cells incorporated BrdU without cytokine stimulation immediately after separation. Figure 1(b) shows representative pictures of CML CD34+ G1/S/G2/M and G0 cells immediately after separation after 1-hour incubation with BrdU. The few G0 cells that incorporated BrdU all showed punctated nuclear labeling indicating an early stage of DNA synthesis while the G1/S/G2/M cells had varied nuclear staining patterns indicating different stages of DNA synthesis.

Without cytokines, the viability of both normal and CML G0 and G1/S/G2/M cells declines rapidly and few viable labeled or unlabeled cells remain after 2-3 days and almost none after 4-5 days. Viability was estimated by the fraction of intact cells excluding trypan blue. However in the presence of 6 or 7 cytokines (KL + FL + TPO, each 50 ng/ml, + G-CSF + GM-CSF + IL-3 ± IL-6, each 10 ng (ml), viability remains excellent (95–100%) and the majority of both normal and CML total CD34+ cells and G0 and G1/S/G2/M cells are induced to proliferate rapidly in liquid culture with average doubling times of ~30 hours. Continuous exposure to BrdU (5 *μ*m) is toxic to both proliferating normal and CML cells, and viability declines rapidly after 2 days so results comparing incorporation of BrdU during continuous exposure of cytokine stimulated G0 cells is limited to the first 48 hours. With stimulation by 7 cytokines, 92% and 94% of CML G0 cells incorporated BrdU during continuous exposure for 24 and 48 hours, respectively, (average of 3 experiments) while the corresponding 24 and 48 values for normal G0 cells were 8% and 72%, respectively. These experiments suggest that the majority of both normal and CML CD34+G0 and G1/S/G2/M cells remain viable and can be stimulated to proliferate with 7 cytokines, but that the CML/G0 cells are more poised than the normal G0 cells to begin proliferating.

### 3.4. Microarray Data Validation

To validate the robustness of our microarray data, we performed quantitative real-time PCR (qPCR) independently on each of four CML CD34+/G0 and two normal bone marrow CD34+/G0 samples that were different from the ones used to generate the microarray data. On each sample, we tested the expression of all the genes represented in Figure 1(c), averaged the results obtained either from the four CML samples or two BM and then calculated the resulting fold change in expression for each gene between the two groups. Since the amount of RNA recovered after the sorting was very limited, we performed a single-step RT-qPCR using the ΔΔct method (the primers used are listed in Table 1, in Supplementary Material available online at doi:10.1155/2011/798592). This way, we were able to use as little as 0.1 ng per reaction enabling us to do four replicates per gene tested. We found that out of fourteen genes tested, which were differentially expressed between the normal and CML/G0 by the microarray, thirteen were confirmed by qPCR (Figure 1(c)).

### 3.5. Signature of CML and Normal Bone Marrow CD34+/G0 Cells

Using a *q* value of less than 0.05 as a threshold, we found 1,204 genes significantly upregulated and 1,133 downregulated in CML/G0 compared to normal BM/G0 cells (for a complete list of the genes see Supplementary Material available online at doi:10.1155/2011/798592). As an additional criterion for selecting the genes significantly differentially expressed, we considered in the analysis only those with differences in expression higher than three folds. This resulted in 292 down- and 192 upregulated genes to be considered. Using this as a starting point, we later extended the analysis to look for genes that could corroborate our initial tentative conclusions, but in this second step, we considered also genes that had at least a two-fold difference and more than one set of probes changed. We have grouped the genes according to their reported functions and discussed the possible significance of the most relevant findings regarding differences between normal and CML CD34+/G0 cells.

### 3.6. Cell Cycle/DNA Replication Related Genes

In the genes linked to cell cycle regulation, we found an almost equal number of them differentially expressed characterizing CML/G0 cells as nonproliferative when compared to normal G0 cells, (e.g., upregulation of MTSS and downregulation of CDC14B), and as proliferative via upregulation of CDC6 and cyclin B2. The most striking difference is in the number of genes upregulated in the CML/G0 cells that are either involved in DNA replication (TOPO2a, RRM2, GINS1 and 2) or are part of the mitotic spindle machinery (MAP9, CETN3, ANLN, DLG7). This subset of CML cells seems to be in a nonproliferative state but essentially ready to enter into the cell cycle upon stimulation, having the machinery for cell division and DNA replication expressed and ready to work. This conclusion is compatible with findings reached independently by another group [16], showing that CML cells are much more easily triggered into cell cycle than their normal counterparts.

### 3.7. Stem Cells and Hematopoietic Stem Cells Markers

Among the most significantly differentially expressed genes that we found between the CML and normal quiescent cells, many of these genes have been reported to be associated with stem cells. The first three genes belonging to this group are Prominin-1 (CD133) [17], ID1 [18], and FLT3 [19]. A second group includes genes that have been found overexpressed in both of two independent studies that have analyzed hematopoietic stem/progenitor cell transcriptomes, HLF, RBPMS (HERMES), GATA3, TNFSF10 (TRAIL), and CRHBP [20, 21].

The third group includes: CD110/MPL, GBP2, SPTBN1, ARG2, BIRC3, CRHBP, HLA-E, HOXA3, HOXB6, SPINK2, NRIP1, PRKCH, RAPGEF2, and TLOC1, all genes that are overexpressed in HSC-enriched populations of bone marrow/cord blood and mobilized peripheral blood cells [20].

Last but not least, we found as differentially expressed MSI2 (Musashi-2), another well-known stem cell marker [22], HES-1 a hematopoietic stem cell marker [22] and IL7, that is, expressed in human adipose-derived stem cells [23].

All these genes are overexpressed in hematopoietic stem/progenitor cells compared to more differentiated ones, but in our microarray, all of them are downregulated in the CML/G0 fraction compared to the normal/G0 fraction. This provides additional evidence that with the methods employed, we are indeed enriching for quiescent hematopoietic stem cells and early progenitors in normal bone marrow but when we apply the same technique to enriching CML quiescent stem and progenitor cells, the latter are in a more advanced stage of differentiation. Because the CML G0 cells were enriched from the blood of patients with highly elevated WBC counts while the normal G0 cells were enriched from the bone marrow, this might offer a partial explanation. However, since previous labeling studies conducted in vivo in CML patients with massive myeloid expansion have shown there is continuous trafficking and exchange of early progenitors as well as maturing cells between the bone marrow, spleen, and blood and in whom the differential counts and proliferative kinetics of the bone marrow and circulating cells were very similar [24], it is more likely that the gene expression results accurately reflect the average results of the entire CML/G0 subpopulation [25].

Another element that supports our conclusion that the CML/G0 cells are more differentiated than the normal G0 cells is that six genes that belong to the Polycomb Repressive Complex 1 (PRC1), respectively, SCML1, PHF1, PCGF3, CBX7, L3MBT1, and 4, and one (EPC2) belonging to the PRC2 group, are downregulated in the CML/G0 fraction (all of them apart from SCML1, with a difference in terms of relative gene expression under the twofold level, so they are not listed in Table 1). The PRC1 and PRC2 complexes belong to the groups of epigenetic regulators and act as gene expression repressors. Both complexes are involved in the maintenance of adult and embryonic stem cells [26]. Downregulation of genes belonging to these two groups in the CML/G0 fraction reinforce the idea that the bulk of quiescent CML cells belong to a more differentiated state than the normal counterpart, as also proposed for blastic phase CML cells in a previous paper by Jamieson [27].

### 3.8. The Majority of the Quiescent CML Cells Overexpress Genes Belonging to the Megakaryocyte-Erythroid (M/E) Lineages

Another series of genes differentially expressed in the CML/G0 fraction enabled us to localize more precisely where the predominant myeloid expansion takes place.CML G0 cells express a series of megakaryocytic (NFE2, TESC and CD41) and erythrocyte markers (CD36, KLF1, TFR2, ANK1, and XK and four different hemoglobin chains (HBB HBQ1 HBD, and HBG1) plus GATA1, a gene whose expression is linked to hematopoietic cell differentiation [28]. The overexpression of GATA-1 is of particular interest since Graf has shown that if GATA-1 is expressed in myeloid cell lines, the cells' phenotype is completely changed to erythroid, probably due to inactivation of the myeloid regulator PU-1 by GATA-1 [29]. An unexpected finding, in view of the more prominent hyperproliferation of megakaryocytic than erythroid cells in CML, is that SMAD7, whose expression promotes megakaryocytic over erythroid differentiation [30], is downregulated in CML/G0 cells.

On the other hand, two key genes promoting lymphoid differentiation (BCL6 and GATA3) are downregulated and a gene expressed in neutrophils (NCF4) is upregulated, as might be expected. These data might be explained by the fact that CML G0 cells are neoplastic cells and therefore exhibit a common cancer-associated characteristic variously termed “lineage infidelity, promiscuity, or ambiguity” [31], a well-known phenomenon occurring in leukemia. It appears that the cells retain the phenotype of the original cells but at the same time deregulate many other pathways characteristic of other hematopoietic lineages, which could also explain why there's expression of fetal hemoglobin chain (HBG1) together with the adult hemoglobins.

In conclusion, it appears that at least the majority of CML/G0 cells overexpress genes usually associated with erythro-megakaryocytic development which is probably correlated with the thrombocytosis frequently seen in patients with CML and also with the spontaneous growth of erythroid colonies in vitro by CML progenitors in the absence of erythropoietin, whereas normal progenitors always require EPO [32].

**Figure 2:**
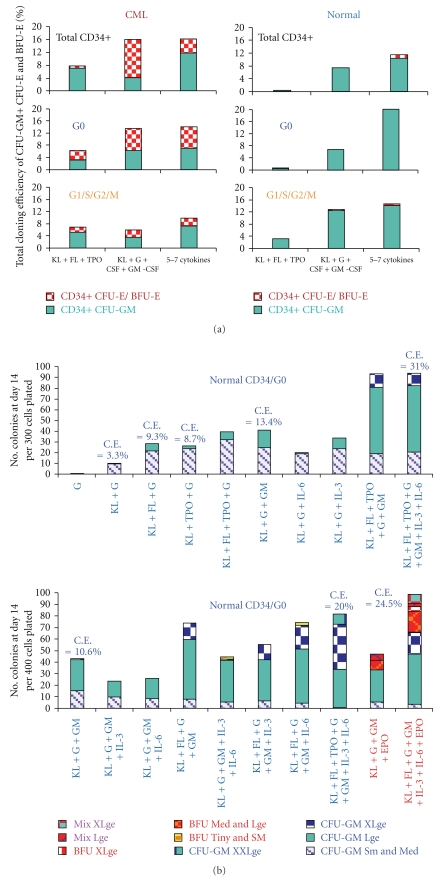

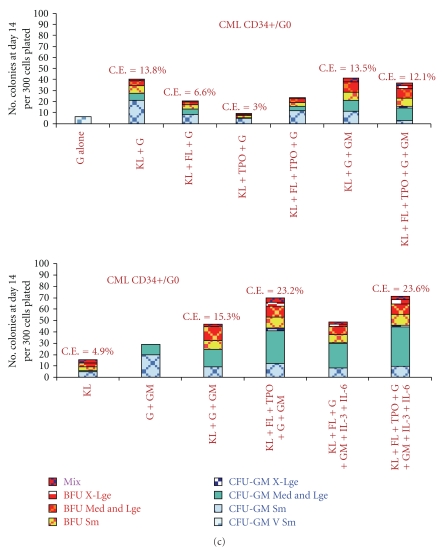
(a) Summary of Multiple Cloning Experiments by normal & CML. Total CD34+ Cells and G0 and G1/S/G2/M fractions. Comparison of average results of multiple cloning experiments (total *n* = 47) comparing total cloning efficiencies of CML and normal total CD34+ cells and G0 and G1/S/G2/M subsets with different cytokine combinations: KL + FL = TPO  *n* = 8 Experiments; KL + G-CSF + GM-CSF *n* = 18; and KL + FL + TPO + G-CSF + GM-CSF ± IL-3 ± IL-6 *n* = 21. (b) Cloning of CD34+ G0 Cells from 2 normal bone marrow samples. Cloning of 2 normal CD34+ G0 cells enriched from fresh bone marrow samples from 2 normal volunteers using the color scale of the estimated colony sizes of numbers of cells per colony according to the standard scale described under methods. The CFU-GM and erythroid (CFU-E/BFU-E) clusters and colonies of all sizes are designated, respectively, CFU-GM and BFU-E on this and subsequent graphs. C.E. = %total Cloning Efficiency. (c) Cloning of 2 CML CD34+ G0 cells enriched from frozen-thawed mononuclear cells obtained from the peripheral blood of 2 CML patients in chronic phase.

Figure 2(a) shows the average results of multiple cloning experiments comparing the cloning efficiencies (C.E.s) of normal and CML total CD34+ cells and the G0 and G_1_/S/G_2_/M subsets. No EPO was added in any of the experiments, but the CML cells consistently produced erythroid colonies without EPO, often including large or X-large BFU-E, and sometimes comprising over half of the total colonies, whereas in the absence of EPO, the normal cells rarely produced any erythroid colonies and then only tiny or very small ones. In almost all experiments, the CML G0 cells generated more and larger erythroid colonies than the G_1_/S/G_2_/M cells. In other experiments not included in Figure 2(a), the addition of EPO to other cytokine combinations greatly augmented further growth of CML erythroid colonies as well as stimulating normal ones. It is also evident in Figure 2(a) that CML total CD34+ and CD34+ G0 cells produced more total colonies than the corresponding normal cells when stimulated by the three early acting cytokines KL, FL, and TPO and to a lesser extend by KL+G− and GM-CSF, again showing the CML progenitors are more easily triggered into cycle than the normal cells. However, there was little difference or the normal G0 cells had higher total C.E.s when the cells were near maximally stimulated with 5–7 cytokines.

Figures 2(b) and 2(c) show typical experiments comparing the cloning results of two normal and two CML G0 cells in more detail. Except for a few tiny or very small CFU-E, normal G0 cells shown in Figure 2(b) produced only CFU-GM colonies without EPO and had the greatest incremental growth of large and extra-large colonies with 5–7 cytokines; with addition of EPO, about 1/4 to 1/3 of the normal colonies were erythroid or mixed, some very large. In marked contrast, the CML G0 cells shown in Figure 2(c), when stimulated in the absence of EPO by all the single cytokine and combinations shown (except G-CSF alone and G-CSF+GM-CSF), produced a mixture of CFU-GM and CFU-E/BFU-E with about a third to one half of the colonies being erythroid or mixed and often very large. 

The maximum total cloning efficiencies of these two G0 samples were ~14–24% after stimulation with 3–7 cytokines, but in other CML and also normal G0 samples total C.E.s of up to 40–42% were observed after stimulation with multiple cytokines. No consistent differences were noted between the maximum total C.E.s between normal and CML CD34+ cells or G0 or G_1_/S/G_2_/M subsets, although there was more variability in the quality of the CML samples.

### 3.9. Transcription Factor Expression

Class I *Homeobox* (*Hox*) genes comprise a family of 39 transcription factors that share a highly conserved DNA binding domain. Since several of them have been shown to play a role in hematopoiesis [33], we looked at their expression profile in our microarray. We found three members of this family under expressed in CML/G0 cells: HOXB3, HOXA5, and HOXA3.

HOXB3 is expressed in the primitive CD34+ population, that is, highly enriched for human hematopoietic stem cells (HSCs), and it is downregulated as the cells differentiate into committed progenitors [34], and together with HOXB4 it is required for normal HSC function. HOXA5 is another gene involved in hematopoietic lineage commitment and maturation, and it seems to act as a repressor of the generation or proliferation of erythroid progenitor cells [34]. Regarding HOXA3, there is not much information about its function in the hematopoietic system. Taken together, the pattern of expression of the HOXA genes in our microarray suggests, again, that the CML CD34+/G0 stem progenitor cells are more mature than the normal counterpart.

Another two transcription factors are differentially expressed in the normal and CML/G0 fractions: NMYC is under expressed and WT1 overexpressed in CML/G0 cells. NMYC is a well-known oncogene found to be expressed in neuroblastomas and retinoblastomas and also in myeloid and lymphoid leukemias [35], but it has not previously been reported to be dysregulated in CML. This gene plays a role in preventing differentiation so its expression is downregulated in cells that progress through more mature stages in order to acquire their final phenotype. NMYC is under expressed in the CML/G0 progenitors so its level of expression may simply reflect their more advanced differentiation and more rapid maturation as previously reported [36]. 

WT1 pattern of expression in normal HSCS is biphasic: high in quiescent CD34+/CD38−, low in committed progenitors, and high again in differentiated cells (CD34−). WT1 also is not expressed in cells expressing erythroid or megakaryocyte markers [37]. So, how to explain the higher expression of WT1 in CML/G0 cells? 

It has been reported that the oncogenic signaling from BCR/ABL can induce WT1 expression [38] and that while in normal mice only a few immature cells in the bone marrow express WT1, when CML is induced the percentage of bone marrow cells expressing WT1 rises considerably [39]. The effect of this gene if expressed in progenitors cells is to keep them in a state in which they are not responsive to differentiation inducing signals. So, it could be that while WT1 tends to keep the CML/G0 cells in an aberrant quiescent state other programs for differentiation are still turned on, such as those inducing differentiation in the M/E lineages, another manifestation of lineage infidelity.

### 3.10. Cancer-Related Genes

As a strategy to identify novel anticancer treatments specific for the CML quiescent population, we looked for genes that were upregulated in the CML fraction and that in the literature had been previously found to have a potential role in other type of cancers. Following these criteria, we found three genes: PVT1, ANXA2, and MARCKS.

MARCKS is a gene important for cell proliferation: it has been reported that while its expression is low in cell lines that are actively proliferating its expression increases when they stop dividing and enter the G0 phase [40]. Additionally, the over expression of MARCKS inhibits proliferation of human tumor-derived choroidal melanoma cells [41]. So its role in CML/G0 cells could be to keep them in an artificial quiescent state and as a consequence protect from the action of cytotoxic drugs. Blocking MARCKS activity would be one step necessary to make these cells respond again to cytokine stimuli and make them reenter the cell cycle. MARCKS is a prominent intracellular substrate of protein kinase C: since different PKC inhibitors have been developed and available (like Enzastaurin, LY317615.HCL), this hypothesis could be tested.

ANXA2 is a lipid- Ca^2+^-actin binding protein that has been reported to be upregulated in different human tumors like hepatocellular and pancreatic carcinoma [42] and acute promyelocytic leukemia [43]. But while in these type of tumors it seems to have a positive role in promoting cancerogenesis and metastasis in other tumors, it seems to slow down their aggressiveness or tumor cell migration, like in osteosarcoma and prostate cancer [44]. The different functional roles of ANXA2 in different malignancies probably reflect its tissue specificity, so without further evidence, it would be premature to suggest it may have a specific role in the CML/G0 population. Nonetheless, it remains a potential target candidate gene.

The PVT1 gene encodes a number of alternative transcripts, but no protein or regulatory RNA products have been found so far; recently, it has been suggested that this region might encodes for different miRNAS where one at least seems to be oncogenic [45]. The amplification of this locus has been shown to contribute to the pathophysiology of ovarian and breast cancer [46], and over expression of PVT1 has been detected in a subset of cases of AML [47] and in other myeloid malignancies. PVT1 role in cancer seems to be that of an inhibitor of apoptosis, so this is another potential target gene that is worth consideration.

These three genes were not identified as overexpressed in a previous work that did gene expression profile comparing total CD34+ CML and normal cells [48], so it is plausible that the different expression levels of these three protein is due to the fact that they belong to the specific quiescent subpopulation of CD34+ CML cells rather than the total CD34+/CML cells.

### 3.11. Prominin-1 (CD133)

The gene most downregulated in CML CD34+/G0 cells compared to normal bone marrow CD34+/G0 cells is prominin-1 or CD133 (–19.6 fold). Prominin-1 is a pentaspan transmembrane glycoprotein which was first isolated in 1997 from plasma membrane protrusions on murine neuroepithelial stem cells and was so named because of its prominent localization in these protrusions (from Latin, prominere) [49]. 

There are two isoforms of the gene, AC133-1 and -2 (which is 27 nucleotides shorter): it was demonstrated that AC133-2 rather than AC133-1 is the predominant transcript expressed in hematopoietic stem cells (HSCs) derived from fetal liver, bone marrow, and peripheral blood as well as in epidermal stem cells, a wide variety of fetal and adult tissues and several poorly differentiated human carcinomas, but not in more differentiated tumors. Based on these findings it was postulated that AC133-2 might serve as a good marker of undifferentiated cells, including stem and progenitor cells present in stem cell niches in multiple fetal and adult tissues.

In contrast, the undeleted AC133-1 transcript originally found in the retinoblastoma cell line was not detectable in fetal liver or kidney or in adult pancreas, kidney, placenta, or brain, but was strongly expressed in fetal brain, suggesting distinct roles for the two isoforms in development and homeostasis of different fetal and mature organs rather than redundancy. Both CD133 isoforms localize to the plasma membrane and have been extensively used alone or in combination with other markers for the identification of stem and progenitor cells from many adult normal tissues and organs [50] including leukemic stem/progenitor cells [51].

Most studies have reported CD133 expression is reduced or lost during later stages of differentiation, but in a recent report [52], using a knock-in lacZ reporter mouse model (*Il*10^−/−^/*CD*133^lacZ^) and immunostaining, CD133 expression was observed in the full spectrum of undifferentiated and differentiated colonic epithelial cells in both mice and humans. Both CD133+ and CD133− metastatic cancer cells formed colonospheres and tumors in NOD/SCID mice that could be serially xenotransplanted, and it was noted the CD133− cells formed more aggressive tumors and were more enriched for phenotypic markers thought to be more typical of cancer initiating cells (CD44+CD24−) than CD133+ cells. In view of our finding that CD133 is downregulated in CML CD34+ G0 cells compared to normal CD34+ G0 cells, it is of particular interest that the authors suggested that its downregulation in aggressive colonic cancer may indicate transformation of primary CD133+ cancer cells into more malignant CD133− metastatic tumors [52].

### 3.12. Colocalization of CD34 and CD133 Antigens 

The CD34 antigen has been widely used as a marker of human stem cells and progenitor cells for both clinical stem cell transplantation and laboratory studies characterizing and comparing normal and leukemic stem and progenitor cells, although it is recognized that a small subset of CD34− cells also have repopulating ability and can give rise to CD34+ cells [53]. Coexpression of CD34 and CD133 has been observed [53], and the cells expressing both antigens were found to have a higher cloning potential than those only expressing CD34 [54]. Numerous studies have led to the recognition that HSCs are concentrated in the Lin-CD34+CD38−/lo fraction [55]. Of particular interest, Wagner et al. observed that the more primitive CD34+CD38− slowly dividing cells expressed higher levels of CD133 than the fast dividing, presumably more mature, CD34+ CD38+ cells [56].

### 3.13. Low Expression of Prominin-1 in CML/G0 Cells May Contribute to Their Altered Lineage Fidelity and Defective Control of Homeostatic Cell Density

Unlike normal bone marrowenriched progenitors which form almost no erythroid colonies or only tiny ones in the absence of EPO as shown in the examples in Figures 2(a), 2(b), and 2(c), CML progenitors often produce erythroid colonies of varying sizes, some very large, after stimulation with various single cytokines or combinations of cytokines without EPO. The production of erythroid colonies by CML progenitors in the absence of EPO is consistent with the previous finding that Bcr-Abl Tyrosine Kinase supports normal erythroid development in erythropoietin-deficient murine progenitor cells [57]. Under EPO deprived conditions, we observed marked inhibition of CML erythroid colony growth by several potent inhibitors of Bcr-Abl [25], thus providing evidence that Bcr-Abl increased Tyrosine Kinase activity cooperates with KL and other cytokine activated pathways in early CML progenitors to reprogram and distort their direction of lineage commitment, which in normal bone marrow and mobilized peripheral blood progenitors, and to a lesser extent in cord blood cells, is more dependent on EPO for production of erythroid cells. The production of erythroid colonies by CML progenitors without EPO at first appears counterintuitive because the major expansion in CML clearly occurs in the granulocyte lineage. However, it is quite compatible with the upregulation of the genes noted earlier that are involved in early erythroid development in CML CD34+ G0 cells compared to normal CD34+ G0 cells. 

**Figure 3:**
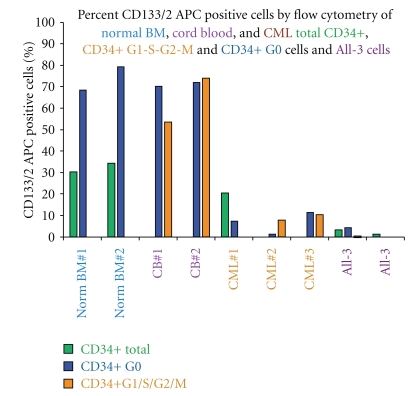
Expression of CD133/2 measured by flow cytometry. Total CD34+ cells, CD34+ G0, and G1/S/G2/M cells enriched from normal bone marrow, pooled cord blood samples, CML chronic phase peripheral blood, and ALL-3 cells and stained with antibody CD133/2 APC (293C3, Miltenyi Biotec). ALL-3 is a p190bcr-abl-driven growth factor independent cell line derived from the blast cells in a pleural effusion of a patient with Ph+ ALL.

**Figure 4:**
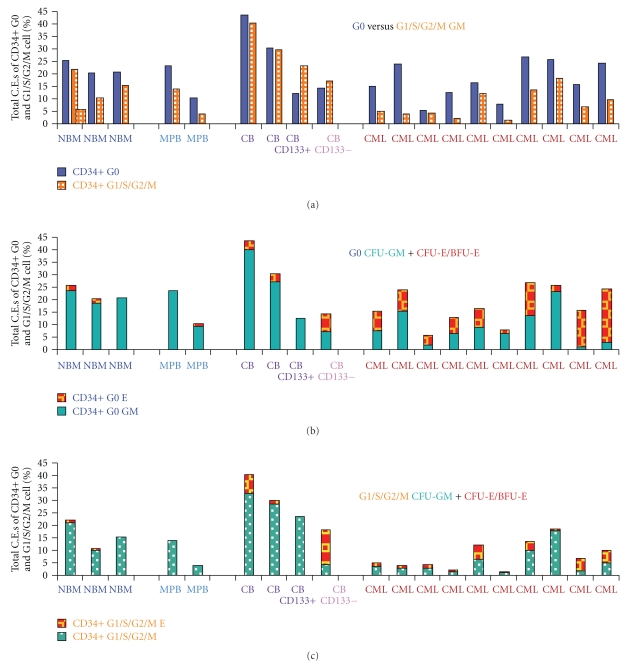
Comparing the total cloning efficiencies (C.E.s) of CD34+ G0 and G1/S/G2M cells from normal bone marrow, normal mobilized peripheral blood, pooled cord blood samples, and chronic phase CML peripheral blood samples. Total Cloning Efficiencies (CFU-GM+CFU-E/BFU-E) at 14-15 days of normal and CML CD34+ G0 and G1/S/G2/M cells after near-maximal stimulation with 5–7 cytokines, all without EPO: (a) CD34+ G0 and G1/S/G2/S cells from 3 normal bone marrow samples, 2 normal mobilized peripheral blood samples, 5 pooled Cord Blood samples, and 10 CML peripheral blood samples. The values for the cord blood CD133+ and CD133- bars shown are the average of 3 separate pooled cord blood experiments. (b) The same CD34+ G0 only samples showing proportions of myeloid (CFU-GM) and erythroid (CFU-E+BFU-E) colonies. (c) The same as B but CD34+ G1S/G2/M cells.

As already noted, prominin-1 (CD133) is the most downregulated gene in CML CD34+ G0 cells compared to normal G0 cells and expression of the CD133/2 antigen is also low in both CML CD34+ G0 and G_1_/S/G_2_/M and Bcr-Abl driven All-3 cells compared to normal bone marrow or cord blood CD34+ G0 and G_1_/S/G_2_/M cells (Figure 3). Unlike normal bone marrow or mobilized peripheral blood CD34+ G0 or G_1_/S/G_2_/M cells which form no or only tiny erythroid clusters on stimulation with 3 to 7 cytokines without EPO (Figures 2 and 4), we observed that pooled cord blood G0 and G_1_/S/G_2_/M cells often produced small, medium, and even large erythroid colonies in the absence of EPO (Figures 4(b) and 4(c)). Figure 4 summarizes a number of representative experiments comparing the total cloning efficiencies (C.E.s) of CD34+ G0 and G_1_/S/G_2_M cells from normal bone marrow, normal mobilized peripheral blood, pooled cord blood samples, and chronic phase CML peripheral blood samples. In all except some of the cord blood samples, the G0 cells had higher total C.E.s and almost always also produced larger GM and erythroid colonies (not shown) than the G1/S/G_2_/M cells (Figure 4(a)), providing additional evidence that they are more primitive.

**Figure 5:**
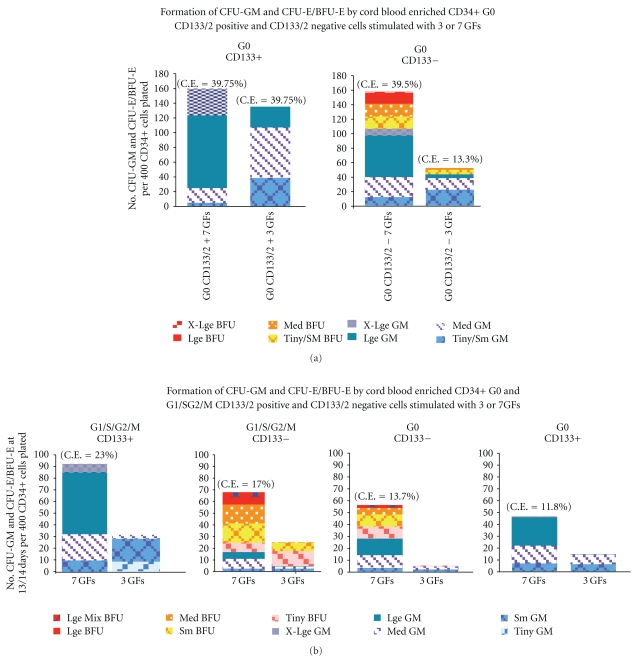
(a) CFU-GM and CFU-E/BFU-E colony formation by CD34+ G0 CD133/2 positive and negative cells, enriched from 3 pooled cord blood samples when stimulated by KL+FL+TPO and the same GFs + G-CSF+GM-CSF + IL-3 + IL-6. No EPO was added. C.E. = % total cloning efficiency. (b) Comparison of three pooled cord blood CD34+ G0 and G1/S/G2/M CD133/2 positive and CD133/2 negative cells ability to form CFU-GM and CFU-E/BFU-E when stimulated by the same 3 or 7 cytokines without EPO as in Figure 5(a). The total C.E.s of the enriched G0 and G1/S/G2/M cells from these pooled cord blood samples are lower than those in Figure 5(a), but again only the CD133/2 negative cells of both the G0 and G1/S/G2/M fractions formed erythroid colonies.

To further examine the effect of CD133 on direction of lineage differentiation, we compared the formation of GM and erythroid colonies by cord blood CD34+ quiescent and proliferating CD133+ and CD133- cells. We observed that cord blood CD34+ G0 133/2+ cells form numerous myeloid colonies, some very large, but no erythroid colonies when stimulated by either 3 GFs or 7 GFs w/o EPO (Figure 5(a)). In contrast, while cord blood CD34+ G0 CD133/2 negative cells had similar total C.E.s with 7 GFs w/o EPO, they formed a mixture of myeloid and erythroid colonies with about a third of the latter being large or extra large. In a similar experiment shown in Figure 5(b) with pooled cord blood G0 and G_1_/S/G_2_/M cells, again the CD133+ cells produced only myeloid colonies and CD133+/2 negative cells from both the quiescent and proliferating fractions formed many erythroid colonies. The cells derived from the pooled cordblood samples shown in Figure 5(b) had lower total C.E.s than those in Figure 5(a), but both the G0 and G_1_/S/G_2_/M CD133/2 negative cells had higher percentages of erythroid and mixed colonies, some very large.

**Figure 6:**
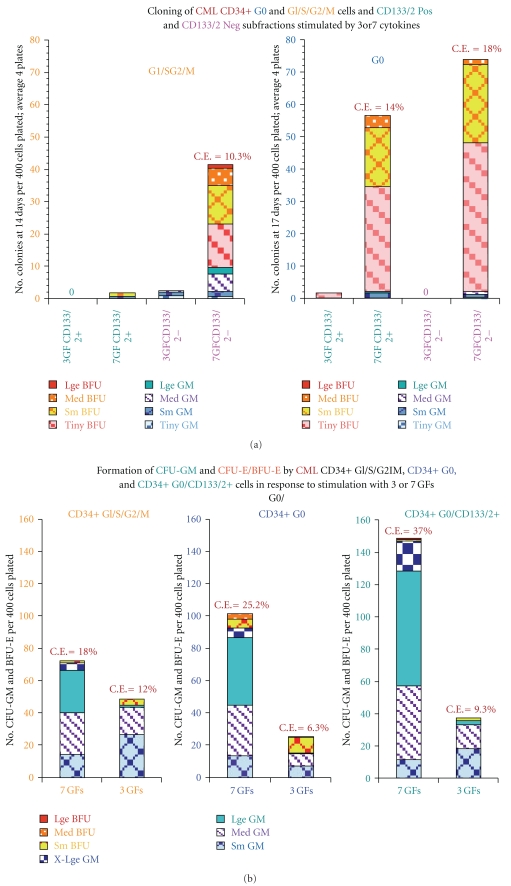

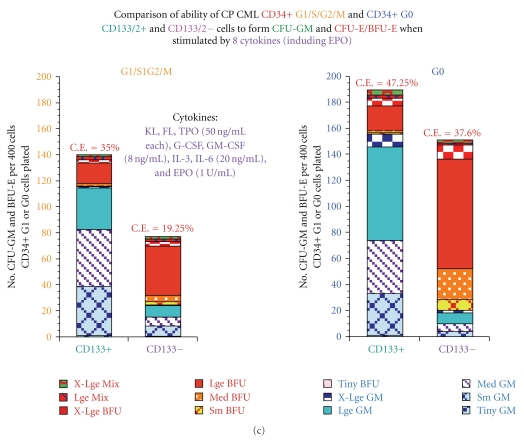
(a) Cloning of CD34+ G0 and G1/S/G2/M cells enriched from pooled peripheral blood samples of 2 untreated chronic phase CML. The total C.E.s were low, and almost all of both the GM and erythroid colonies were tiny, small, or medium sized with very few large colonies and with very few or no colonies after stimulation with 3 growth factors (KL+FL+TPO), pointing out the variable quality of the CML samples received. In this case, both the G0 CD133 positive and negative fractions formed almost entirely small erythroid clusters and colonies with 7 GFs, but only the CD133 negative G1/S/G2/M cells produced colonies, including some GM colonies. (b) Colony formation by G0 and G1/S/G2/M cells enriched from the peripheral blood of a chronic phase CML patient. The sample produced GM colonies with fairly high C.E.s following stimulation with 3 or 7 cytokines without EPO. With additional enrichment of the G0 cells (G0 CD133/2+) the C.E.s further increased by about a third, with production of almost entirely GM colonies. (c) Maximum stimulation of G0 and G1/S/G2/M cells enriched from the peripheral blood of a chronic phase CML patient.The G0 cells had a high total C.E. after stimulation with 8 cytokines shown, including EPO. Even with addition of EPO, while both the G0 and G1/S/G2/M CD133 negative cells produced predominately large, X-large erythroid, and mixed colonies, the CD133 positive fractions produced mainly GM colonies, but also about 20% large and X-large BFU-E plus mixed colonies.

Whereas the results clearly demonstrate that CD133/2+ cord blood-derived G0 and G_1_/S/G_2_/M cells in the absence of EPO only form GM colonies and the CD133/2− cells form a mixture of erythroid and GM colonies, the distinction is less consistent and the results are more variable in comparable CML cells. Figure 6(a) illustrates colony formation by CML G0 and G_1_/S/G_2_/M cells with relatively low C.E.s enriched from pooled samples of two chronic phase CML patients. Both G0 CD133/2 positive and negative cells formed almost entirely tiny or small erythroid colonies when stimulated with 7 cytokines while only the CD133/2 negative G_1_/S/G_2_/M cells formed any colonies, again mostly tiny or small erythroid ones, but with some GM colonies. The three cytokines, KL+FL+TPO, stimulated growth of very few or no colonies. Figure 6(b) shows an example of colony formation by CD34+ G0 and G_1_/S/G_2_/M cells enriched from another chronic phase CML patient in which the cells formed mostly GM colonies, but with further separation of G0 CD133/2+ cells, the total C.E. increased by about a third and there were higher proportions of large and X-large GM colonies and almost no even very small erythroid clusters. An example of a third CML cloning experiment is shown in Figure 6(c) in which chronic phase CML G0 cells had very high total C.E.s when EPO was added to the 7GFS, especially the CD133+ cells. As usually observed, the G0 cells had higher C.E.s than the G_1_/S/G_2_/M cells. Both the quiescent and proliferating CD133 negative cells formed mostly erythroid colonies while the CD133 positive cells formed predominantly GM colonies even in the presence of EPO, although about 17% of the colonies were large or X-large BFU-E and mixed colonies. 

In summary, based on our observations so far, it appears that Prominin-1, the product of the gene most downregulated in CML CD34+ G0 cells, is also underexpressed on the surface of both CML CD34+ G0 and G_1_/S/G_2_/M compared to normal bone marrow or cord blood CD34+ G0 and G_1_/S/G_2_/M cells (Figure 3). Cord blood CD34+ G0 133/2 positive cells form numerous myeloid colonies, some very large, but no erythroid colonies when stimulated by either 3 GFs or 7 GFs without EPO (Figures 5(a) and 5(b)). In contrast, under the same conditions, cord blood CD34+ G0 CD133/2 negative cells form a mixture of myeloid and erythroid colonies with some of the latter being large or X-large. 

The role of (downregulated) CD133 in CML CD34+ G0 and G_1_/S/G_2_/M cells is presently less clear and appears to be more complex. The CML G0 cells in Figure 6(a) had low C.E.s and both the CD133 positive and negative fractions produced almost entirely tiny and small erythroid colonies even when stimulated by 7 cytokines, while only the G_1_/S/G_2_/M CD133− cells produced a substantial number of colonies, mostly erythroid. In the experiment shown in Figure 6(b), the CML G0 CD133+ fraction when stimulated with the same 7 GFs without EPO increased the total C.E. by about a third compared to the total G0 cells, but the colonies were almost entirely GM, whereas the total G0 cells produced about 10% small- and medium-sized erythroid colonies. Finally as shown in Figure 6(c), the CML CD133+ G0 cells when stimulated by 7 GFs + EPO had one of the highest C.E.s (47.25%) we have observed with mostly large and X-large GM colonies, but also about 17% large and X-large BFU-E and mixed colonies, while the CD133− cells formed mostly erythroid colonies, mostly large or X-large. 

The above observations suggest that expression of CD133 in both normal and CML progenitors favors GM differentiation while CD133 negative cells produce a mixture of erythroid and GM colonies. However, the precise function of CD133 is still very uncertain and confounded by the necessity to use multiple cytokines to stimulate growth. CML and cord blood progenitors are less dependent on EPO in producing erythroid cells than normal bone marrow progenitors, but addition of EPO markedly shifts the cells toward erythroid differentiation. Overall, the results suggest that Prominin-1 may have an important role in determining the direction of lineage commitment, especially in cord blood and CML progenitors. We found no consistent difference in the numbers or sizes of the colonies produced by either normal or CML quiescent or proliferating CD133+ or CD133− cells, although some experiments suggested enrichment of one or the other might further enhance their overall proliferative potential.

### 3.14. Correlations of Gene Expression and Surface Antigen Expression

An attempt was made to see if it was possible to correlate changes in gene and surface antigen expression as measured by flow cytometry in subpopulations of normal and CML cells, but because of the small number of cells usually recovered it was only possible to do limited surface phenotyping in a minority of enriched samples.

**Figure 7:**
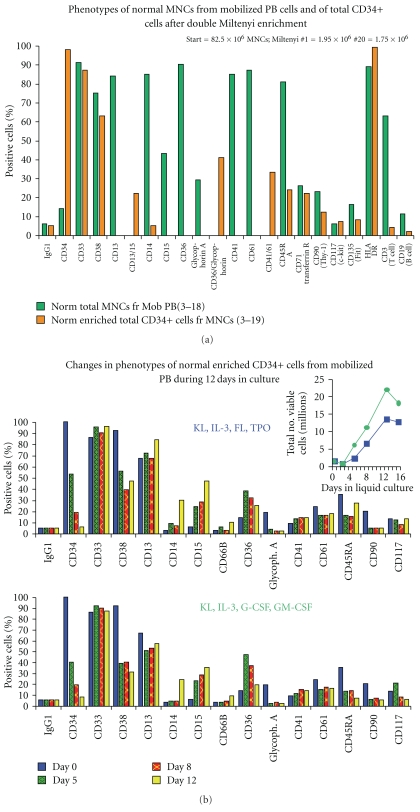
(a) Expression of surface antigens determined by flow cytometry of normal mononuclear cells (MNCs) after Ficoll Hypaque separation from a normal frozen-thawed mobilized peripheral blood sample compared to expression of total CD34 cells after two enrichments of the same MNCs on Miltenyi columns as described in Methods. Insufficient numbers of CD34+ cells were recovered to permit comparison of additional antigens. (b) Changes in cell surface antigen expression in total CD34+ cells enriched from a sample of frozen thawed mononuclear cells from normal mobilized peripheral blood during 12 days of culture. The cells were stimulated to proliferate with the two combinations of cytokines shown in the concentrations stated under Methods.

**Figure 8:**
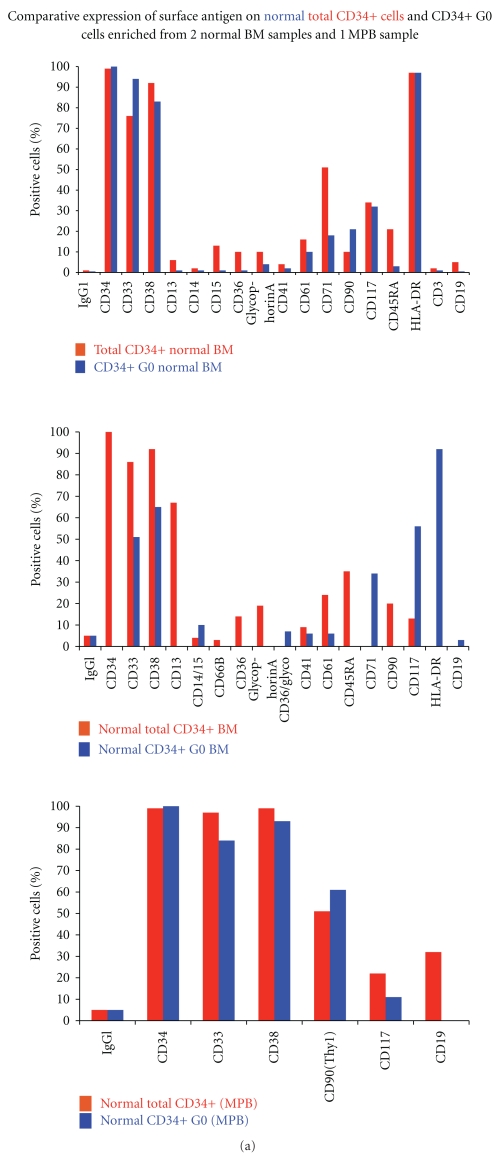

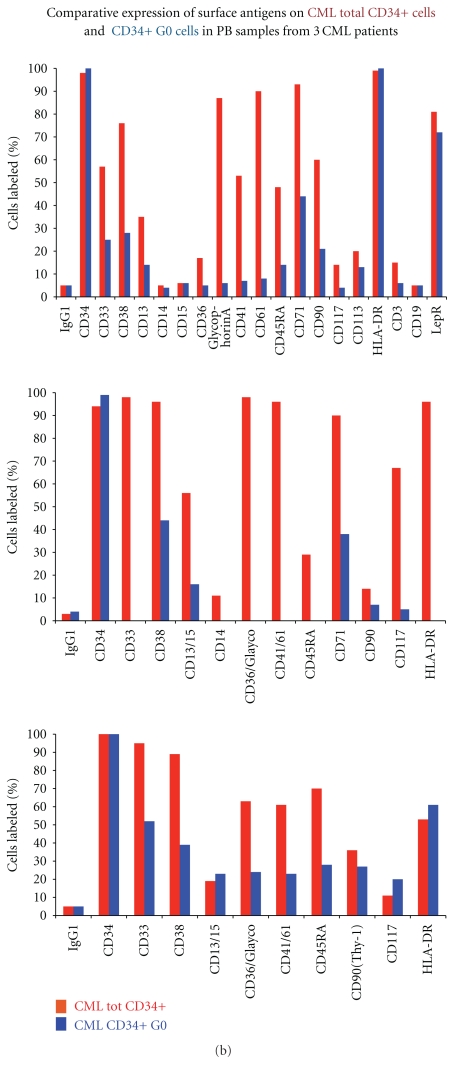
(a) Comparative cell surface antigen expression of 3 normal total CD34+ and CD34+ G0 cells enriched from 2 fresh normal bone marrow samples and one frozen-thawed normal mobilized peripheral blood (MPB) sample. (b) Comparative expression of cell surface antigens of 3 CML total CD34+ and CD34+ G0 cells enriched from 3 frozen-thawed mononuclear cell fractions enriched from peripheral blood samples from 3 CML patients. The sample in the upper chart was from a patient in early accelerated phase, while the lower 2 were from patents in chronic phase.

**Figure 9:**
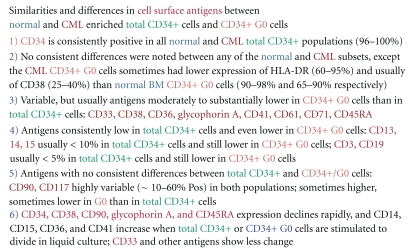
Summary of the most consistent similarities and differences observed in cell surface antigen expression in multiple experiments between normal and CML enriched total CD34+ and CD34+ G0 cells.

Examples of representative experiments showing similarities and differences in expression of surface antigens are shown in Figures 7 and 8, and a summary of the overall results of multiple surface phenotyping experiments is given in Figure 9.

 In these and other studies, there were insufficient CD34+ total cells or subsets to compare additional surface antigens other than those shown. As illustrated in Figure 1(a) and Table 1, the CD34+ G_1_/S/G_2_/M cells are usually much more numerous than the CD34+ G0 cells so their phenotype is very similar to that of the total CD34+ cells (not shown).  Figure 7(a) shows a typical study comparing surface markers of normal mononuclear cells from mobilized peripheral blood and the enriched total CD34+ cells from the same sample showing marked enrichment of CD34+cells and reduced expression of antigens expressed on more differentiated cells, including CD14, CD45RA, CD90, CD3, and CD19. Figures 8(a) and 8(b) show comparisons of cell surface antigen expression of normal and CML total CD34+ and CD34+/G0 cells in several typical experiments. As would be expected, in most but not all normal and CML samples, expression of CD33, CD38, and CD45RA were reduced in the CD34+ G0 cells compared to the total CD34+ cells. Figure 7(b) shows the changes that occur in surface antigens during 12 days in liquid culture when normal CD34+ cells are stimulated to proliferate by two combinations of 4 cytokines. In the latter experiment, following stimulation with both cytokine combinations, CD34, CD38, Glycophorin A, CD90, and CD45 RA expression rapidly declined and this usually occurred even faster in stimulated CML CD34+ cells (not shown). Most surface markers associated with granulocyte/macrophage, erythrocyte or megakaryocyte differentiation increased both in normal and CML CD34+ cells (e.g., CD 14, 15, 36, 41), although CD61 declined slightly. As is evident from comparing individual experiments, there was considerable variation in both normal and CML individual samples, but we have nevertheless attempted to provide an overall summary in Figure 9 of the most consistent findings in multiple experiments of the similarities and differences in surface antigen expression between normal and CML total CD34+ cells and CD34+ G0 cells and when the latter are stimulated to proliferate. Overall, we did not detect any consistent differences between normal and CML total CD34+ cells or in their G0 and G_1_/S/G_2_/M subsets except that the CML G0 cells usually had lower percentages of cells expressing CD38 and sometimes of HLA-DR than the normal G0 cells, but the results were not consistent enough to draw any firm conclusions.

## 4. Discussion

This study was initiated based on the assumption that quiescent CML cells and early progenitor cells may differ in their pattern of gene expression from comparable normal cells and that if critical differences could be identified, they would help to reveal the underlying molecular mechanisms responsible for the excessive overproduction of the CML population. It was further hoped that some vulnerable differences would be discovered that would be susceptible to targeting by highly selective drugs, since it was assumed that the quiescent cells would be largely unaffected by existing BCR-ABL inhibitors such as Imatinib and Dasatinib as well as other drugs active against proliferating cells. So far, there has been only one other study attempting this [58], which was performed using the same methods but using fewer samples (five CML and two normal) and an older microarray platform with considerably less genes represented (14,500 versus 38,500). 

Because at least 10^5^ cells were required for each sample for microarray analysis and the enriched CD34+ G0 cells comprised less than 0.02% of the starting cell populations, it was difficult to obtain sufficient cells, especially patient samples, to carry out the study. We initially collected 10 CML samples, but 2 had insufficient RNA so only 8 are included. We also performed microarray analyses on CD34+ G0 and G_1_/S/G_2_/M cells enriched from cord blood and normal G-CSF mobilized peripheral blood samples, but excluded them from the final analysis because some of the results differed from cells enriched from normal bone marrow samples and we considered the latter to be more appropriate normal controls. Both the normal and CML quiescent and proliferating fractions consisted of 96–100% CD34+ primitive blast cells and less than 1% of CD34+ G0 cells incorporated BrdU, so almost all of the latter were either in G0 or early G_1_. Because there are no definitive markers to distinguish stem cells from early progenitor cells, it is unknown what percentage of each was present in the CD34+ G0 fractions, but we presume the majority of cells in both the normal and CML fractions were progenitors in various stages of development. Ideally, we would have preferred to also compare normal and CML stem cells, but were unable to do this because of a lack of clear stem cell markers and insufficient cell numbers.

Some of the genes that were differentially expressed in normal and CML/G0 cells revealed a number of interesting findings which correlate nicely with some of the biological and functional abnormalities previously observed in CML cells. These findings include the following. (i) In keeping with the gene expression data, normal and CML quiescent G0 cells are more highly enriched in primitive cells than the proliferating G_1_/S/G_2_/M cells. (ii) The CML G0 cells are in a slightly more advanced stage of development than the normal G0 cells, and as previously reported by Graham et al. [58], the CML CD34+ G0 cells are more similar to the CD34+ proliferating cells than are the normal G0 and G_1_/S/G_2_/M fractions. (iii) In keeping with their more advanced stage of development and their upregulation of genes involved in DNA replication or part of the mitotic spindle machinery, CML/G0 cells are more poised to begin proliferating than normal G0 cells and are more sensitive to growth stimulation by single cytokines or combinations known to act on early progenitors and stem cells. While normal and CML/G0 cells are almost equally responsive to stimulation by multiple cytokines, the CML cells are triggered into cycle more rapidly. (iv) Once CML quiescent progenitors are stimulated by cytokines to begin proliferating, they undergo further differentiation and maturation more rapidly than normal quiescent progenitors, but both granulopoiesis and erythropoiesis are usually less efficient than in normal hematopoiesis as shown in cloning experiments in which the CML cells form many more small CFU-GM, CFU-E, and BFU-E compared to normal progenitors [14, 32]. (v) Whereas normal CD34+ cells form almost entirely granulocyte/monocyte clusters and colonies in clonogenic experiments when stimulated by cytokines in the absence of erythropoietin, CML CD34+ G0 cells consistently spontaneously form a combination of GM and erythroid colonies in the absence of EPO. The gene expression data clearly shows that CML/G0 cells have marked overexpression of genes associated with development of the erythrocyte and megakaryocyte lineages, and Graham et al. noted similar findings [58]. (vi) Prominin-1 (CD133) is the gene most downregulated in CML G0 cells, and there is lower expression of CD133 on the surface of these cells. Cord blood G0 CD 133+ cells form only GM colonies without EPO while CD133− cells form a mixture of GM and erythroid colonies. The downregulation of CD133 appears to be associated with the spontaneous formation of erythroid colonies by CML progenitors in the absence of EPO, but its precise role remains to be better clarified. It has been known for many years that both normal and neoplastic cell populations contain significant numbers of “resting” or quiescent cells that are considerably less sensitive to the damaging effects of irradiation, cytotoxic drugs, and other injurious agents than are proliferating cells [59, 60]. The dormant state is a protective mechanism that is of crucial importance in enhancing a population's probability of survival under adverse conditions, and early on the concept that dormant cancer cells are important obstacles to curability was widely recognized by both basic and clinical scientists [15, 25, 61]. If one accepts the premises that almost all lethal cancers originate in adult stem cells or early progenitors functioning as stem cells, that these cells are essential for initiation, maintenance, and expansion of the cancers, and that a large fraction of these cells, like normal stem cells, reside in a quiescent state in which they are resistant to most therapies, then it is obviously important to better understand their derivation and properties, to determine how normal and cancer quiescent stem cells may differ, and to look for ways to develop specific targeted therapies based on these differences.

Activation of quiescent cells following a mitogenic stimulus by serum, cytokines, or other factors is highly complex and involves the coordinated and selective induction of expression and repression of hundreds of genes including specific cyclins, cyclin-dependent kinases (CDKs), and protein kinase inhibitors (PKIs). Many cell cycle genes are transcriptionally silent in quiescent cells, and they express only a limited number of cytokine receptors, and recent studies have shown that siRNAs and microRNAs are also involved in repressing gene transcription and translation in quiescent cells. When one considers the staggering complexity of all of the cytokines and other regulatory factors and cellular interactions that determine whether a quiescent stem cell residing in its protected niche is going to divide while simultaneously deciding what kinds of cells to produce, it is hardly surprising that our present understanding of the mechanisms regulating stem cell behavior is very incomplete. In our analysis, it appears that in many cases, clusters of presumably related genes that are differentially expressed are all associated with a particular stage of development or function, suggesting that their common dysfunction in CML G0 cells may involve aberrant co-regulation.

Most authorities agree that the HSC population is heterogeneous, but it is still uncertain at what level of development the system permits changes to occur in phenotype and functionality, or when the differentiation hierarchy becomes fixed. Circumstantial evidence for both normal and leukemic stem cells favors a heterogenic model in which there is a continuum of stem and early progenitor cells with gradually declining potentials for self-replication, pluripotency, and other stem cell properties, but that some cells may also exhibit flexibility in responding to different stochastic influences in their developmental milieus. Some degree of reversibility may also exist whereby early progenitors can retain or reassume more primitive stem cell properties if needed, such as the ability for more extensive self-replication in order to replace or supplement damaged stem cells. However, it is likely that significant reversibility is restricted to early progenitors and that once they have become committed to differentiate along a particular lineage, it is doubtful that they can revert to functioning as stem cells. With regard to cancer stem cells, some years ago we postulated that most leukemias originate in “limited stem cells” which have more limited pluripotency than normal primitive HSCs, but retain sufficient self-replicating potential to initiate a lethal leukemia [11]. Many current researchers now agree, although some prefer to call them leukemia or cancer “initiating cells” to distinguish them from true HSCs.

While cells undergoing differentiation and maturation can become temporarily arrested or slowed in their progression through other phases of the cell cycle under certain conditions (e.g., hypoxia, increased cell density, exposure to toxins, cytotoxic drugs, irradiation, or other damaging agents), it is usually only stem cells and primitive progenitors that remain in a quiescent state for extended periods under normal steady-state conditions. Once progenitors become firmly committed to differentiation and maturation, serial cloning studies conducted in vitro [32] and cytokinetic labeling studies with ^3^H-thymidine conducted in vivo [24, 62] have shown that both normal and leukemic cells usually proceed to undergo a variable but limited number of maturation divisions to produce terminally differentiated cells (which may be highly abnormal in leukemia and other malignancies), and which are incapable of reverting to regain significant self-renewal or other essential stem cell properties. While our in vivo ^3^H-thymidine labeling studies were less extensive in patients with lymphomas or solid tumors growing in ascetic form [63, 64], in cases in which it was possible to distinguish neoplastic cells in differing states of maturity, it appears that once the neoplasticcells become committed to maturation, if the environment is suitable, they usually continue to divide but are only capable of a limited number of divisions before dying spontaneously and are incapable of reproducing the disease. Overall, our in vivo labeling studies strongly suggest that the number of dormant cancer stem and progenitor cells continue to increase as the population of cancer cells expands and that they, thus, constitute a progressively greater obstacle to curative therapy in many types of cancer [11, 25, 65].

The constitutive tyrosine kinase activity of the p210^bcr-abl^ protein causes abnormal phosphorylation of regulatory proteins in numerous interacting signaling pathways [3, 25, 66, 67]. The overall signaling networks altered by Bcr-Abl are highly complex [68], indeed reaching a level of complexity that some observers have likened to Heisenberg's uncertainty principle in quantum mechanics. Nevertheless, although the specific signaling changes responsible for each of the biological abnormalities that have been described are still incompletely defined, it is highly likely that the faulty signaling disrupts multiple interactive networks that normally tightly regulate the orderly well-coordinated processes of proliferation, differentiation, and maturation in normal hematopoiesis. This misregulation can probably explain all the abnormalities observed in early-stage CML including the initial overproduction of GM progenitors, the imbalanced lineage apportionment, the inefficiency of production of both granulocytes and erythrocytes, and all the other more subtle dysplastic morphological, biochemical, and functional changes that have been described [25]. 

Gleevec and some of the newer Bcr-Abl inhibitors are highly effective in globally inhibiting the increased tyrosine phosphorylation of multiple proteins involved in these signaling pathways [25, 69–71] and since Bcr-Abl is usually the sole mutation in early stage CML, the progenitors are restored to near normal behavior when the kinase is adequately inhibited. At higher concentrations, the Abl TKI inhibitors are lethal to both fresh primary CML progenitors and Bcr-Abl-driven cell lines while at still higher concentrations, they also kill normal progenitors and cell lines not driven by Bcr-Abl, the exact normal: CML lethal concentration ratios depending on the particular cells and inhibitors.

However, as suggested by the usual relatively slow induction of remissions in CML patients over the course of weeks or months, it is doubtful if the BCR-ABL inhibitors when administered in clinically tolerable doses actually kill many of the proliferating CML progenitors and precursors. Rather, by inhibiting Bcr-Abl's constitutive tyrosine kinase activity, they at least transiently restore the cells to functioning more normally, and in so doing the CML progenitors cease excessive cell production, presumably by reacquiring the ability to respond properly to quorum sensing signals that ensure maintenance of normal homeostatic cell density limits. Meanwhile, the later CML progenitors which are already committed to differentiation continue to proceed through a limited number of maturation divisions and then die as terminally differentiated cells, just as do normal cells. After the body burden of leukemic cells has been sufficiently reduced, the residual normal stem cells are released from the CML cells' (poorly understood) inhibitory effects and resume production, usually resulting in a complete hematologic or cytogenetic remission. The BCR-ABL inhibitors are clearly a very important advance since they are able to induce durable remissions in the majority of CML patients in chronic phase, but they are not usually curative since most patients relapse if therapy is discontinued, probably because quiescent CML stem/progenitor cells are not killed by the drugs and are able to reproduce the disease [9, 72]. Several mechanisms of resistance have also been well described as noted earlier [7, 8]

Thus, while Gleevec and other Bcr-Abl TKIs are very effective in the early stage of CML in largely eliminating the majority of proliferating Ph+ progenitor and precursor cells, more attention should be given to seeking ways to selectively kill the quiescent leukemia stem and progenitor cells. While our own studies so far have not revealed any specific vulnerable targets, it is important that the search be continued. The alterations in gene expression described here must be confirmed in a larger number of patients, and if possible with further technological advances, in still more highly enriched populations of early progenitors and stem cells. As the search proceeds, the significance of some of the differences in gene expression reported here may become clearer and eventually lead to discovery of new ways to selectively kill the quiescent CML stem and progenitor cells. For a number of reasons, it has become increasingly difficult to obtain large enough samples of CML cells to carry out the rigorous procedures required to isolate sufficient numbers of highly enriched stem and progenitor cells for further studies, so this is another important issue that must be addressed.

In a broader sense, it is perhaps even more important to design similar therapeutic strategies for other types of cancer. Much of the recent development of anticancer drugs has been directed towards producing different classes of drugs that block segments of one or more signaling pathways that are known to be dysregulated by the particular initiating mutation(s) commonly found in different types of cancer. However in advanced malignancies it is often difficult to distinguish the importance of the primary causative mutation(s) compared to that of secondary or still later (passenger) mutations and the situation may become further complicated by multiple epigenetic changes. A huge number of new drugs of different classes are now available, but few cause complete or durable remissions and they are almost never curative. Greater emphasis should therefore be placed on more clearly identifying and whenever possible selectively targeting the primary driving mutations in the cancer stem or early progenitor cells in early stage disease, and also in developing new strategies to selectively kill the quiescent stem or progenitor cells that escape most current therapies.

## Supplementary Material

Table.1 Primers used for qRT-PCR.

## Figures and Tables

**Table 1:** Numbers of G0 and G1/S/G2/M cells recovered from highly enriched normal and CML CD34+ population. In the upper part of the table are shown the data showing the recovery of cells from normal bone marrows and in the lower part the recovery of cells from the CML patients. In bold is the average recovery of each fraction relative to the CD34+ starting population. The last column shows the percentage of blasts from each patient at the moment of sample collection, and the asteriks indicate the patients in accelerated phase (more than 5% of blasts). Cells recovered from BM #2 were not used due to the insufficient number of cells recovered. Legend: starting CD34+ cells in millions (S-CD34+), CD34+ G1/S/G2/M cells recovered (R-CD34+ G1/S/G2/M), CD34+ G1/S/G2/M cells recovered as % of starting CD34+ cells (%G1/S/G2/M), CD34+ G0 cells recovered (R-G0), and CD34+ G0 cells recovered as % of starting CD34+ cells (% G0).

Normal BM	S-CD34+	R-G1/S/G2/M	%G1/S/G2/M	R-G0	%G0	% blasts
1	2.55	400,000	15.3	50,000	1.96	—
2	0.5	64,000	12.8	19,400	3.9	—
3	5.85	1,000,000	17.1	400,000	6.84	—
4	2.8	370,000	13.2	73,000	2.56	—
5	3.6	1,170.00	32	238,000	6.6	—

		**M** **e** **a** **n** = 3.06%	**M** **e** **a** **n** = 18.0%		**M** **e** **a** **n** = 4.3%	

CML patient	S-CD34+	R-G1/S/G2/M	%G1/S/G2/M	R-G0	%G0	% Blasts

1	6	580,000	9.6	100,000	1.6	2%
2	3.5	387,000	11	123,000	3.51	1%
3	0.9	230,000	25.5	42,000	4.6	<3%
4	1.7	320,000	18.8	74,800	4.4	3%
5	4.25	310,000	7.29	150,000	3.52	1%
6	4.59	300,000	6.53	150,000	3.26	*18%
7	0.85	158,000	18.5	13,500	1.58	*14%
8	3.45	719,000	20.8	68,700	1.99	*8.5%

	**M** **e** **a** **n** = 3.15%		**M** **e** **a** **n** = 14.7%		**M** **e** **a** **n** = 3.2%	

**Table 2:** List of genes, discussed in the paper, with significantly differential gene expression in CML CD34+/G0 cells compared to normal bone marrow. The fold change is linear and positive values mean genes more expressed in CML and negative values vice versa. In the case that for one gene there is more than one set of probes significantly differentially expressed, we reported the highest value among them. For the complete list of genes, see Supplementary Material available online at doi:10.1155/2011/798592.

Gene symbol	Fold change (CML/BM)	Gene symbol	Fold change (CML/BM)
*Apoptosis*		HLA-E	−2.81
FAS	+2.9	HOXA3	−4.44
MX1	−3	HOXB6	−4.97
BIRC3	−3.1	SPINK2	−10.71
MALT1	−4.44	NRIP1	−2.41
NALP1	−2.29	PRKCH	−6.43
*Cell proliferation*		RAPGEF2	−2.6
MTSS1	+5.58	TLOC1	−1.77
DLG7	+3.71	HES-1	−8.45
ANLN	+3.56	*Differentiation*	
CCNB2	+3.34	CD36	+19.7
CDC6	+3.28	XK	+8.7
CETN3	+3.16	Klf1	+6.97
MAP9	−3.51	ITGA2B (CD41)	+6.51
MAP9	−3.51	GATA1	+4.0
CDC14B	−5.18	NFE2	+3.3
*DNA replication*		TESC	+4.5
RRM2	+5.57	ANK1	+3.9
GINS1	+3.49	HBQ1	+3.2
TOPO2A	+3.34	HBD	+18.4
GINS2	+3.33	HBG1	+17.3
Growth factors/cytokines		HBB	+13.4
IL7	+3.26	NCF4	+3.9
*Stem cell markers*		BCL6	−4.88
PROM1	−19.75	GATA3	−4.99
HLF	−18.08	TFR2	+5.5
GBP2	−3.85	LEPR	+20.6
FLT3	−6.6	*SMAD7	−8.7
SPTBN1	−4.97	*NMYC	−4.1
RBPMS	−5.93	*Transcription factors*	
GATA3	−4.99	HOXB3	−3.23
MPL (CD110)	−6.11	HOXA5	−3.76
TNFSF10 (TRAIL)	−3.07	WT1	+3.7
MSI2	−4.18	*Cancer genes*	
ID1	−7.32	PVT1	+7.0
ARG2	−2.92	ANXA2	+5.75
BIRC3	−3.88	MARCKS	+8.21
CRHBP	−14.75		
