# Supplementary material for: Gene Expression Differences between Enriched Normal and Chronic Myelogenous Leukemia Quiescent Stem/Progenitor Cells and Correlations with Biological Abnormalities
Source: J Oncol. 2011 Feb 23;2011:798592. doi: 10.1155/2011/798592 (PMC3062978; doi:10.1155/2011/798592)
Supplement: Supplementary file 1 — Table.1 Primers used for qRT-PCR. [file 798592.f1.zip › 798592.f1/Supp dat.pdf]

CTGCACCACCAACTGCTTAG	GAPDH Forward
GGCCATCCACAGTCTTCTG	GAPDH Reverse
GCCTGGAGGAGCTGTACC	CPI-17 F.
GATTTTCCGGCTTCTCTCCT	CPI-17 R.
GCAGACACGCGGGAGAAAAC	ADAMTS3 F.
TGCGCACCAGGACCACATT	ADAMTS3 V.
GCCGACATTGCCTCCTACCAG	NEFH F.
GCCAAAGCCAATCCGACACTCT	NEFH F.
CCGTCCTGTCCTGGCTGCTC	RRM2 F.
CCGGCGGCGTGTTCTCC	RRM2 R.
ATGTTCCGAACCCCAAGAAT	Leptin Receptor F.
AGTTGGCACATTGGGTTCAT	Leptin Receptor R.
CTCTTCGCTACCCAGGTGAC	GATA3 F.
GTAAAAAGGGGCGACGACTC	GATA3 R.
CTCAGTGTTGGTGTGGTGATG	CD36 F.
CACAAATTGGTTTGTGCTTGA	CD36 R
GTGACCTCAGCCCCTCACT	Integrin alpha 2b F.
CGGGATGGCTACAATGACA	Integrin alpha 2b R.
CATTTGTGGGGTGAATTCCT	Hemoglobin-D F.
AGCTGCACTGTGACAAGCTG	Hemoglobin-D R.
CCCTGATGACCTGAAGGATG	Hepatic leukemia factor F.
TGGCGATCTGGTTCTCTTTC	Hepatic leukemia factor R.
TTTCAAAATTTCTGCATTCACG	Anphiregulin F.
TCATGGACTTTTCCCCACA	Anphiregulin R.
ATGGCAACTCTGATCCCAAC	Protocadherin9 F.
ATCGAGTGACCCAAAACCAA	Protocadherin9 R.
TGGTGATCAAAATATCTGATCTTACC	CRHBP F.
GCAGCTCCACAAAGTCTCCT	CRHBP R.
GCTGATGTTGAAACTGCTTGA	CD133 F.
TTGCTCCTGGATTTGGAAAG	CD133 R.
TGAAAGTCTTCAGAATGGAAGGA	Ki67 F
GGTCAGAAGAGAAGCTAGATCTTGAG	Ki67 R.

Table.1 Primers used for qRT-PCR
